# Gender Differences in the Association between Sleep Duration and Body Composition: The Cardia Study

**DOI:** 10.1155/2010/726071

**Published:** 2009-11-12

**Authors:** Marie-Pierre St-Onge, Suzanne Perumean-Chaney, Renee Desmond, Cora E. Lewis, Lijing L. Yan, Sharina D. Person, David B. Allison

**Affiliations:** ^1^New York Obesity Research Center, St. Luke's/Roosevelt Hospital, 1090 Amsterdam Avenue, suite 14D, New York, NY 10025, USA; ^2^Department of Biostatistics, University of Alabama at Birmingham, Birmingham, AL 35294, USA; ^3^Division of Medicine, University of Alabama at Birmingham, Birmingham, AL 35294, USA; ^4^Division of Preventive Medicine, University of Alabama at Birmingham, Birmingham, AL 35294, USA; ^5^Department of Preventive Medicine, Northwestern University, Chicago, IL 60611, USA; ^6^Department of Health Economics and Management, Guanghua School of Management, Peking University, Beijing 100871, China

## Abstract

Sleep duration has been inversely associated with body mass index (BMI). We examined the relationship between self-reported sleep duration and BMI, waist circumference, and percent body fat in Black and White individuals from the CARDIA study. Box-Tidwell regression models were adjusted for age and race (Model 1), additional lifestyle and demographic variables (Model 2), and physical activity (Model 3). There were significant interactions between sleep and gender for the main outcome variables. In men, there was a trend for an inverse relationship between reported sleep duration and BMI in Model 2  (*β* = −0.20, *P* = .053) but not model 3  (*β* = −0.139, *P* = .191). In women, inverse relationships were observed between sleep duration and BMI (*β* = −0.294, *P* = .005) and waist circumference (*β* = −0.442, *P* = .059), in Model 2. These associations became nonsignificant in model 3 (BMI: *β* = −0.172, *P* = .084; waist circumference: *β* = −0.161, *P* = .474). Our results are consistent with previous findings that sleep is associated with BMI and other body composition variables. However, the relationship between self-reported sleep duration and body composition may be stronger in women than in men.

## 1. Introduction

Physical activity and dietary patterns have received much attention in attempts to explain the rise in obesity of past decades; however, many other factors have been identified that may have contributed to the obesity epidemic that we observe today [1]. One contributor receiving much interest recently is sleep debt. In a 2004 survey, approximately 63% of adults reported sleeping 7-8 h/night and almost 30% averaged less than 6 h/night [2]. In the Sleep Heart Health Study, mean sleep duration was reportedly slightly over 7 h/night in both men and women [3]. In the Coronary Artery Risk Development in Young Adults (CARDIAs) study, young adults were getting approximately 6 hours of sleep/night [4] with Black men and women sleeping, on average, about 1 h/night less than White men and women (about 5.5 versus 6.4 h/night).

Such short reported sleeping times may be of concern in the context of an obesity epidemic since several studies have found that sleep duration is inversely associated with body mass index (BMI, in kg/m^2^) [5–8]. Data from the National Health and Nutrition Examination Survey I (NHANES I) showed that young adults getting <7 hours of sleep/night had higher BMI and were more likely to be obese than those sleeping 7 h/night or more [9]. Similar findings are observed in children and adolescents [10–13]. Furthermore, data from the Nurses' Health Study show greater weight gain in women sleeping less than 7 h/night compared with those sleeping at least 7 h/night [14]. Women sleeping 7-8 h/night also had the lowest risk for major weight gain (≥15 kg over 16 years) and sleeping ≥7 h/night was not associated with an increased risk of developing obesity in normal weight women whereas sleeping 5 or 6 h/night was. Similarly, in the Quebec Family Study, those who reported sleeping 5-6 h/night gained approximately 2 kg more over a 6-year follow-up period than those who reported sleeping 7-8 h/night [15]. 

Despite the large number of epidemiological studies linking obesity and short sleep duration [16], there is a paucity of data carefully examining the relationship between body composition and sleep duration in adults. Strengths of our cohort study include the presence of waist circumference, and percent body fat data from dual energy X-ray absorptiometry (DXA) as well as the inclusion of both Black and White individuals. Waist circumference contributes independently to the prediction of visceral adipose tissue [17] and it is therefore of interest to assess its relationship with sleep duration. The main objective of this study was to examine relationships between self-reported sleep duration and BMI, waist circumference, and percent body fat in the CARDIA cohort. We hypothesized that short sleep duration would be associated with higher BMI, waist circumference and percent body fat. Secondary objectives were to determine whether body composition also showed similar relationships with sleep duration between Black and White adults and between men and women. This study is important since it is one of the few studies to examine the impact of sleep duration on body composition indices in both Black and White men and women. It is interesting to note that Black individuals tend to be more overweight and obese than Whites [18] and also tend to sleep less [4]. It is possible that sleep duration may have a greater impact on obesity status in Blacks than Whites, yet this has not been examined. Similarly, gender differences in the impact of sleep duration on obesity status have been noted in children but not extensively studied in adults [19]. 

## 2. Methods

Data for the analyses reported here are from the CARDIA Study, a cohort study established to examine the determinants and evolution of cardiovascular disease risk factors in young Black and White adults. A total of 5115 men and women, aged 18–30 years, were recruited from 4 centers in 1985-1986: Birmingham, AL (University of Alabama at Birmingham), Chicago, IL (Northwestern University), Minneapolis, MN (University of Minnesota), and Oakland, CA (Kaiser Permanente Oakland). Subjects were examined at baseline and years 2, 5, 7, 10, and 15. At the time of preparation of this manuscript, year 20 data were not available and only cross-sectional analyses are reported here. Furthermore, sleep was assessed only at examination Year 15, preventing us from assessing longitudinal relationships with body composition data from prior examination years. All subjects provided written informed consent at each visit and study protocols were approved by the Institutional Review Board of each institution. Details of the recruitment and study procedures are reported elsewhere [20]. We certify that all applicable institutional and governmental regulations concerning the ethical use of human volunteers were followed during this research.

For the present study, we examined the relationship between sleep duration and indices of body composition from examination at year 15, when subjects were 33–45 years of age. Of the initial cohort of 5115 subjects, 3672 (72%) returned for the year 15 visit. We excluded subjects who had low (<18.5) and extremely high (>45) BMI, implausible sleep data (≥15 h/night), those who reported drinking more than 3 standard deviations above the mean alcoholic beverage consumption (>30 drinks per week), women who were pregnant or breastfeeding, and subjects with missing data. Our final sample size was 3473 subjects for the anthropometric analyses (BMI and waist circumference). DXA was only done at the Birmingham and Oakland sites on a subsample of the study population, allowing analyses examining the relationship between sleep duration and body fat in 320 participants [21]. 

### 2.1. Demographics and Lifestyle Information

Demographic characteristics, age, sex, race, education level, and income level, were self-reported on standardized questionnaires. The information used in this study was collected at the year 15 examination. Education level was determined by obtaining the highest grade or year of regular school completed and was included in the models as a continuous variable (range 1–20+ years). Income level was categorized as total combined family income for the past 12 months and coded ordinally (1 = < $5000, 2 = $5000–11 999, 3 = $12 000–15 999, 4 = $16 000–24 999, 5 = $25 000–34 999, 6 = $35 000–49 999, 7 = $50 000–74 999, 8 = $75 000–99 999, and 9 = ≥ $100 000). Data are presented with groups 1–4 combined (<$25 000), 5–7 combined ($25 000–74 999), and 8-9 combined (≥$75 000). Leisure-time physical activity and work-related physical activity were assessed using a validated interview-administered questionnaire.

### 2.2. Body Composition Measurements

Weight (to the nearest 0.2 kg) and height (to the nearest 0.5 cm) were recorded with subjects wearing light clothing and without shoes. BMI was calculated as weight in kg divided by the square of height in meters. Waist circumference was measured at the level of minimal abdominal girth (to the nearest 0.5 cm) and recorded as the average of 2 measurements. Body composition was measured using DXA QDR-2000 scanner (Hologic, Inc., Waltham, MA). Daily phantom scanning was done to ensure consistent data acquisition throughout the study.

### 2.3. Sleep Questionnaire

A standardized sleep questionnaire was administered at 15 years of followup. The questionnaire included the question: “During the past month, how many hours of actual sleep did you get at night? (this may be different than the number of hours you spend in bed).” The answer to this question was used to determine self-reported sleep duration. 

### 2.4. Statistical Analyses

Descriptive statistics consisted of frequencies, percentages, means, and standard deviations. The relationship between continuous predictors and anthropometric outcomes was assessed with regression models and for categorical variables with the Cochran-Armitage trend test.

To initially assess the association of sleep with body composition variables, Box-Tidwell regressions were conducted [22]. Nonlinear relationships were noted for both sleep duration and age with the four dependent variables (weight, BMI, waist, and percent body fat). The Box-Tidwell procedure was utilized to identify an appropriate power transformation of the independent and dependent variables to linearize relationships. To adjust for the nonlinear relationship, all four dependent variables were log-transformed. Once the transformations were selected in the Box-Tidwell regressions, the new transformed variables were included in ordinary multiple linear regressions. The transformed variables were rescaled by linear transformation to their original mean and variance for ease of interpretation and presentation. Table 1 shows the respective transformations used for sleep and age by gender. There was a significant interaction between gender and sleep for the main outcome variables and therefore the models were stratified by gender. 

The appropriate transformed variables were used in three regression models for each dependent variable. In Model 1, the impact of sleep on the dependent variable was adjusted for age and race. Model 2 was further adjusted for lifestyle (smoking, alcohol intake) and demographic (education level, income) factors. These lifestyle variables are routinely included in models assessing the impact of one behavior on body composition [14]. Factors that did not significantly impact the dependent variable for both men and women were removed for the analyses. Model 3 was additionally adjusted for physical activity level. The beta (*β*) coefficients reported are unstandardized estimated regression coefficients calculated using predictor variables after they have been transformed (as described above) and then rescaled via linear transformations to their original mean and variance to ease interpretation. For instance, a 1-hour increase in sleep changes the dependent variable by *β* units. All models were tested for interactions between race and sleep and no significant interactions were found. Therefore the estimates reflect the combined racial groupings. A two-tailed *α* level of 0.05 was considered statistically significant. Data were analyzed using SAS software for Windows version 9.1 (SAS Institute Inc., Cary, NC).

## 3. Results

A total of 1585 men and 1888 women were included in the main analyses. For the analyses using data from DXA measurements, the sample included 159 men and 161 women. Table 2 shows subject characteristics by gender and race for the main analyses. Most comparisons were statistically significant. In general, women were older than men, weighed less, had higher percent body fat, drank less alcohol and smoked less, reported sleeping longer, and were more inactive than men. Generally, Blacks were younger, heavier, had lower education and income level, reported sleeping less, were less physically active, and smoked more than Whites.

Sleep duration tended to be inversely associated with BMI in men in Model 1 (*β* = −0.20, * P* = .053) and after controlling for other lifestyle and physiologic variables in Model 2 (*β* = −0.198, * P* = .068) (Table 3). In Model 3, after including physical activity, sleep was not significantly associated with BMI in men (*β* = −0.139, * P* = .191). In model 3, race, smoking, alcohol intake, education level, family income, and physical activity level were all significantly associated with BMI. In women, the inverse association between sleep duration and BMI was significant after controlling for age and race (*β* = −0.332, * P* = .002) and other lifestyle and physiologic variables (*β* = −0.294, * P* = .005). However, after physical activity was added to the model, the association between sleep and BMI became nonsignificant (*β* = −0.172, * P* = .084). In women, in model 3, age, race, education level, family income, working full time, and physical activity level were all significantly associated with BMI. 

There was no statistically significant association between sleep duration and waist circumference in men, although the magnitude and direction of the sample slope was similar to that observed in women (Table 4). In women, waist circumference was significantly inversely associated with sleep duration in model 1 (*β* = −0.543, * P* = .022). This relationship was no longer statistically significant when we further controlled for other lifestyle and demographic variables, but was similar in magnitude and nearly statistically significant in model 2 (*β* = −0.442, * P* = .059) but not in model 3 (*β* = −0.161, * P* = .474). Percent body fat was not significantly associated with sleep duration in neither men nor women. 

## 4. Discussion

This is one of the few studies to examine the relationship between sleep and body composition variables beyond body weight and BMI in adults. Furthermore, it is the only study to date to examine the relationship between sleep and body composition in a bi-racial population of Blacks and Whites. As such, this is the first study to examine whether sleep duration is related to waist circumference and percent body fat in Black and White adults.

Few studies to date have examined the relationship between waist circumference and sleep in adults. In the Better Health for Better Hong Kong campaign, waist circumference was inversely related to sleep duration [5], in agreement with our data in women. In men, although the association was not statistically significant, the magnitude and direction of the association between waist circumference and sleep duration was similar to that observed in women. However, in a sample of older Spanish adults, waist circumference was not related to sleep duration [23]. Results from the Quebec Family Study also showed larger waist circumference in men and women who reported sleeping <7 h/night compared to those who reported sleeping 7-8 h/night [24]. It is possible that the age difference between the Spanish cohort and our young adult cohort is responsible for the different results. This relationship between waist circumference and sleep duration is of importance since well-known metabolic disturbances are associated with increased waist circumference [25]. 

In our study, BMI and waist circumference were inversely associated with sleep duration in women before including physical activity in our model. In men, associations tended to be in the same direction and of similar magnitude, but were not statistically significant. This gender difference has not been consistently reported in adults. In fact, no gender effect on the association of sleep and BMI was observed in an elderly Spanish cohort [23], a primary care U.S. sample [26], or the Quebec Family Study [24]. However, in a sample of Southern France, sleep duration was found to be related to BMI in women but not men [27]. Gangswisch et al. [9] have previously reported that BMI was higher in men and women who slept less than 7 hours compared to those who slept 7 hours or more only in individuals age 32–49. This age group includes the age group of our cohort (33–45 years). In the NHANES data, gender differences were also observed in the association between BMI and sleep duration, with women being progressively more likely to be obese as sleep duration was reduced below 7 h/night whereas men were more likely to be obese with 6 or fewer hours of sleep/night. In our study, the association between BMI and sleep duration was significant in women but a trend in men. Our data add to the body of literature suggesting that sleep may have a slightly greater impact on body composition in adult women than in men. This potential gender difference in the relationship between sleep and adiposity deserves further exploration, especially since discordant data have also been observed in children and adolescents [10, 11, 28, 29]. Previous metabolic studies have been conducted in young men only [30, 31] or in a study with few women [32]. Future metabolic studies should be done in women to determine if they have a different hormonal response to short sleep duration than men.

Our finding that percent body fat was not significantly associated with sleep duration is novel. One previous study has examined the relationship between this body composition measure and sleep duration in adults [24]. In our cohort, we did not find any significant association between these two variables. In the Quebec Family Study, Chaput et al. [33] found lower percent body fat in men and women who reported sleeping 7-8 h/night compared to those reporting 5-6 hours of sleep per night. It is possible that our sample size was not sufficient to detect an association. In fact, only 320 subjects participated in the DXA measurement, leaving few participants within each race-gender category. In comparison, the Quebec Family Study had more than twice our sample size with only one racial group.

In this study, adding physical activity to our models attenuated or abolished the relationship between sleep duration and body composition. We can speculate that sleep may in part act on body composition via changes in physical activity. It is possible that adults who sleep less are too tired to be physically active or those who sleep very long have too little time to be physically active. In fact, in our data, physical activity tended to increase with increasing sleep duration in women (data not shown). Moreover, being physically active can improve sleep quality and duration [34, 35] such that physical activity and sleep can be positively related. 

Conservation of energy is a well-known principle that applies to obesity research. In this principle, energy is neither created nor lost. Therefore, in order to lose weight, one must expend more energy than is consumed or consume less energy than is expended; to gain weight, one must expend less energy than is consumed or consume more energy than is expended. It thus follows that our hypothesis explaining the relationship between sleep duration and body composition must (short of variations in energy partitioning) be due to an effect of sleep on energy expenditure or energy intake. If one controls for energy intake (food consumption) and energy expenditure (not equivalent, but closely related to physical activity), much of the variance in body composition variables is accounted for. This was the case when we added physical activity level to our models. In addition, food intake may be increased in those who sleep less. An increase in food intake with shorter sleep duration may result from various factors; longer hours awake create more time for eating, and hunger may increase with shorter sleep duration. Increased hunger ratings have been reported with short sleep duration and these have been related to reduced leptin and increased ghrelin levels [33]. Furthermore, in a recent report from the Quebec Family Study, the relationship between sleep duration and adiposity measures disappeared after controlling for leptin levels [24]. In fact, 88% of short sleepers had low leptin levels, confirming a previous clinical study showing that short sleep decreases leptin concentrations [33]. Dietary information was not taken at Y15 in the CARDIA study and we could not include this variable in our models.

Our study has several limitations. First, our sleep data are self-reported. Sleep was objectively measured in a subsample of our cohort [38]. In that study it was shown that the correlation between self-reported sleep duration and actigraph-measured sleep duration was 0.47. Furthermore, there was a systematic overestimation of sleep duration that was greater in short sleepers than in those sleeping more than 7 h/night. Also, self-reported sleep duration by obese individuals tended to be more closely related to actigraph-measured sleep duration than lean individuals, who tended to overestimate their sleep duration in self-reports relative to the actigraph measurement. Thus, obese individuals may provide more accurate self-reports of their sleep duration than nonobese individuals and nonobese individuals over-report their sleep times. These data suggest that longer sleep hours would falsely include a greater proportion of lean individuals than obese individuals. This might then lead one to erroneously conclude that BMI decreases with increasing sleep duration even if this were not actually the case. In fact, Knutson and Lauderdale [36] have reported that the odds ratio for overweight in adolescents using time-diary sleep times was not significantly affected by sleep duration whereas the odds ratio for overweight was significantly increased with reduced sleep duration when sleep duration was self-reported. On the other hand, Nixon et al. [37] found a significant inverse association between actigraphy-measured sleep duration and overweight/obesity in 7-year old children. Similarly, Taheri et al. [7] found a significant U-shaped relationship between sleep duration and BMI when sleep was measured using polysomnography. More research is needed to determine the degree of error introduced by biased sleep reporting into the association of sleep duration and obesity.

Finally, our data show that the relationship between sleep duration and body composition is not affected by race but rather differs according to gender. In fact, women show a more consistent effect of sleep duration on body composition than men. Our data thus suggest that short sleep duration may be a risk factor for overall and abdominal obesity in young Black and White women. However, our overall small sample size, self-reported measures of sleep, and the cross-sectional nature of our data do not allow us to make definitive conclusions on the impact of gender on the relationship between sleep duration and obesity. More studies are needed to examine the gender difference on the relationship between sleep and obesity and consideration must be given to objectively-measured sleep duration. 

## Figures and Tables

**Table 1:** Transformations for sleep and age by gender.

Dependent Variable	Men		Women	
	Sleep	Age	Sleep	Age
BMI	0.25 power	Square root	Square root	0.25 power
Waist circumference	Log	Log	Log	0.75 power
Percent Body Fat	Square root	None	Log	0.25 power

**Table 2:** Participant characteristics by gender and race.

Characteristic	White men (*n* = 897)	Black men (*n* = 688)	White women (*n* = 962)	Black women (*n* = 926)
	Mean (sd)	Mean (sd)	Mean (sd)	Mean (sd)

Age (years)	40.67 (3.36)	39.47 (3.73)*	40.79 (3.36)	39.67 (3.84)*
Weight (lbs)	193.95 (34.21)	199.85 (41.49)*	158.0 (35.20)	183.14 (39.99)*
BMI (kg/m^2^)	27.65 (4.34)	28.59 (5.24)*	26.23 (5.57)	30.66 (6.26)*
Waist circumference (cm)	93.67 (11.59)	92.88 (12.55)	80.76 (12.85)	88.74 (13.49)*
Body fat (%)^1^	26.88 (6.26)	24.40 (7.82)*	38.25 (9.70)	43.81 (7.46)*
Education level (years)	15.69 (2.66)	13.71 (2.21)*	15.82 (2.40)	14.09 (2.19)*
Alcohol intake (drinks/wk)	4.92 (6.20)	4.69 (6.91)	3.16 (4.84)	1.90 (4.08)*
Sleep duration (h/night)	6.65 (1.00)	6.26 (1.37)*	6.84 (1.09)	6.28 (1.48)*
	N (%)	N (%)	N (%)	N (%)
Family income				
< $5000-$24 999	61 (6.8)	145 (21.6)*	75 (7.9)	246 (27.1)*
$25 000-$74 999	361 (40.3)	345 (51.7)	431 (45.0)	478 (52.6)
$75 000- ≥ $100 000	474 (52.9)	178 (26.7)	450 (47.1)	186 (20.3)
Full-time work				
Yes	801 (89.3)	539 (78.7)*	559 (58.1)	695 (75.5)*
No	96 (10.7)	146 (21.3)	403 (41.9)	226 (24.5)
Difficulty paying for basics				
Very hard	12 (1.3)	20 (2.9)*	20 (2.1)	40 (4.4)*
Hard	13 (1.5)	24 (3.5)	28 (2.9)	35 (3.8)
Somewhat hard	84 (9.4)	99 (14.5)	108 (11.2)	176 (19.2)
Not very hard	787 (87.8)	541 (79.1)	806 (83.8)	667 (72.6)
Physical activity				
Inactive	34 (3.8)	25 (3.6)*	47 (4.9)	94 (10.2)*
	144 (16.1)	69 (10.0)	177 (18.4)	184 (20.0)
Moderately active	391 (43.7)	298 (43.3)	419 (43.6)	430 (46.7)
	205 (22.9)	125 (18.2)	191 (19.9)	100 (10.9)
Very active	120 (13.5)	171 (24.9)	127 (13.2)	112 (12.2)
Current smoking				
Yes	155 (17.3)	228 (33.3)*	150 (15.6)	233 (25.2)*
No	741 (82.7)	458 (66.7)	811 (84.4)	691 (74.8)
Sleep quality				
Very bad	5(0.6)*	19 (2.8)	12 (1.3)	24 (2.6)
Fairly bad	127 (14.2)	70 (10.2)	138 (14.4)	150 (16.3)
good	268 (30.0)	241 (35.1)	289 (30.1)	296 (32.2)
Fairly good	319 (35.7)	219 (31.9)	326 (34.0)	287 (31.3)
Very good	174 (19.5)	137 (20.0)	195 (20.2)	161 (17.6)

^1^ *n* =*  *320

*P  < .05 for comparison between race, within gender category.

**Table 3:** Multivariate models for predictors of body mass index in the CARDIA study stratified by gender (*β*,95%*  *CI)^1^.

Variable	Men			Women		
	Model 1	Model 2	Model 3	Model 1	Model 2	Model 3
Sleep (h)	−0.20	−0.20	−0.14	−0.33	−0.29	−0.17
	(−0.40, 0.002)	(−0.41, 0.01)	(−0.34, 0.07)	(−0.53, −0.13)*	(−0.50, −0.09)*	(−0.37, 0.02)
Age (yrs)	0.003	0.004	−0.02	0.12	0.13	0.12
	(−0.06, 0.07)	(−0.07, 0.07)	(−0.09, 0.05)	(0.05,0.20)*	(0.06,0.20)*	(0.05,0.19)*
Race	−0.72	−1.17	−1.40	−4.49	−3.28	−3.14
(White versus Black)	(−1.23, −0.22)*	(−1.72, −0.62)*	(−1.94, −0.86)	(−5.03, −3.94)*	(−3.88, −2.68)*	(−3.72, −2.57)*
Current smoker		−1.54	−1.54		−0.47	−0.50
(Yes versus No)		(−2.18, −0.89)*	(−2.17, −0.91)*		(−1.17, 0.23)	(−1.17, 0.18)
Number		−0.06	−0.06		−0.09	−0.06
drinks/week		(−0.10, −0.03)*	(−0.09, −0.02)*		(−0.15, −0.03)*	(−0.11, 0.0005)
Education (yrs)		−0.12	−0.15		−0.32	−0.30
		(−0.23, −0.009)*	(−0.26, −0.04)*		(−0.44, −0.19)*	(−0.42, −0.18)*
Income		0.20	0.21		−0.33	−0.25
(ordinal 1–9)		(0.04,0.35)*	(0.06, 0.36)		(−0.47, −0.18)*	(−0.40, −0.11)*
Work full-time		−0.06	0.18		0.65	0.71
(Yes versus No)		(−0.83, 0.71)	(−0.57, 0.93)		(0.08,1.21)*	(0.17, 1.25)
Physical activity			−1.04			−1.57
			(−1.27, −0.81)*			(−1.80, −1.33)*

^1^Beta (*β*) coefficients reported are unstandardized estimated regression coefficients calculated using predictor variables after they have been transformed and then rescaled via linear transformations to their original mean and variance.

*P < .05.

**Table 4:** Multivariate models for predictors of waist circumference in the CARDIA study stratified by gender (*β*,95%*  *CI)^1^.

Variable	Men			Women		
	Model 1	Model 2	Model 3	Model 1	Model 2	Model 3
Sleep (h)	−0.40	−0.35	−0.15	−0.54	−0.44	−0.16
	(−0.90, 0.11)	(−0.89, −0.18)	(−0.67, 0.36)	(−1.01, −0.07)*	(−0.90, 0.02)	(−0.60, 0.28)
Age (yrs)	0.22	0.24	0.17	0.38	0.40	0.36
	(0.05,0.38)*	(0.08,0.41)*	(0.008, 0.33)	(0.21,0.54)*	(0.24,0.56)*	(0.21, 0.52)
Race	0.98	0.39	−0.36	−8.31	−5.69	−5.39
(White versus Black)	(−0.22, 2.18)	(−0.93, 1.72)	(−1.63, 0.91)	(−9.53, −7.09)*	(−7.01, −4.37)*	(−6.65, −4.13)
Current smoker		−2.93	−2.91		0.25	0.18
(Yes versus No)		(−4.48, −1.39)*	(−4.39, −1.43)*		(−1.32, 1.83)	(−1.32, 1.69)
Number		−0.10	−0.07		−0.16	−0.08
drinks/week		(−0.19, −0.006)*	(−0.16, 0.02)		(−0.29, −0.03)	(−0.21, 0.04)
Education (yrs)		−0.43	−0.51		−0.72	−0.68
		(−0.70, −0.16)*	(−0.77, −0.25)*		(−1.00, −0.45)*	(−0.95, −0.42)
Income		0.35	0.45		−0.79	−0.62
(ordinal 1–9)		(<0.001,0.70)*	(0.12, 0.79)		(−1.11, −0.46)*	(−0.93, 0.30)
Physical activity			−3.33			−3.58
			(−3.88, −2.78)			(−4.11, −3.06)*

^1^Beta (*β*) coefficients reported are unstandardized estimated regression coefficients calculated using predictor variables after they have been transformed and then rescaled via linear transformations to their original mean and variance.
